# Comparison of Postoperative Analgesia Between Intrathecal Nalbuphine and Intrathecal Fentanyl in Infraumbilical Surgeries: A Double-Blind Randomized Controlled Trial

**DOI:** 10.7759/cureus.58503

**Published:** 2024-04-17

**Authors:** Seetharaman Jananimadi, B.T. Arish, Balraj Hariharasudhan, Segaran Sivakumar, George K Sagiev, Eashwar Neelakandan

**Affiliations:** 1 Anesthesiology, Pondicherry Institute of Medical Sciences, Pondicherry, IND; 2 Anesthesiology, Broomfield Hospitals, Mid and South Essex NHS Foundation Trust, Chelmsford, GBR

**Keywords:** spinal anesthesia, local anesthetic, hemodynamic response, 0.5% bupivacaine, postoperative analgesia, intrathecal opioids, fentanyl, nalbuphine, additives

## Abstract

Introduction: Spinal anesthesia is a widely used regional anesthesia technique for surgeries below the umbilicus, but postoperative analgesia is of major concern due to the relatively short duration of the local anesthetic. Various drugs were used as an additive to local anesthetic to prolong the duration of postoperative analgesia. This study aims to compare the efficacy of nalbuphine and fentanyl as an intrathecal additive along with local anesthetic.

Methodology: A total of 166 patients aged between 18 and 65 years belonging to the American Society of Anesthesiologists (ASA) I and II undergoing elective infraumbilical surgeries were included in the prospective double-blind randomized controlled trial. The patients were allocated into two groups of 83 each. Group N was given 2.5 mL of 0.5% bupivacaine + 1 mg of nalbuphine (0.5 mL), and group F received 2.5 mL of 0.5% bupivacaine + 25 mcg fentanyl (0.5 mL). Both groups were compared for postoperative analgesia, onset and duration of both sensory and motor blockade, intraoperative hemodynamics, and side effects.

Results: All demographic data, hemodynamic parameters, and side effects were not statistically significant among the two groups. However, other parameters, such as the mean duration of analgesia, which was 267.27 ± 172.099 minutes in group N and 161.35 ± 14.957 minutes in group F; meantime for the onset of sensory blockade, which was 3.94 ± 1.769 minutes in group N and 5.94 ± 0.929 minutes in group F; onset of complete motor blockade, which was 7.10 ± 1.858 minutes in group N and 11.61 ± 1.218 minutes in group F; duration of motor blockade, which was 182.57 ± 13.011 minutes in group N and 112.53 ± 7.389 minutes in group F; and mean time taken for two-segment regression, which was 118.20 ± 12.61 minutes in group N and 113.72 ± 8.84 minutes in group F, were all comparable between the two groups.

Conclusion: Nalbuphine was found to be more efficacious for prolongation of postoperative analgesia with better hemodynamic stability.

## Introduction

Spinal anesthesia has stood the test of time in being the safest and gold standard anesthetic technique and has ever since been in use for surgeries performed below the level of the umbilicus until the present. The development of intrathecal anesthesia has seen leaps and bounds of growth, both in the variety of drugs used and the adjuvants added to enhance the performance from its origins from the first ever used cocaine intrathecally [1]. The intrathecal anesthetic technique would be advantageous in par that it drastically reduces the risk of respiratory complications and incidence of coagulation abnormalities alongside offering early mobility and reduced postoperative stay [2].

Several conventional local anesthetic agents such as lidocaine, bupivacaine, tetracaine, and chloroprocaine have been used traditionally; however, the newer ones such as levobupivacaine and ropivacaine have also shown considerable promise with respect to the quality and intensity of anesthetic blockade [3]. Nevertheless, the greatest challenge posed was to offer an excellent maximization of the duration of analgesia in the postoperative period, which could be achieved with adjuvants in addition to the shorter-acting local anesthetics during spinal anesthesia.

Additives are drugs that, when co-administered with local anesthetics intrathecally, tend to improve the quality of anesthesia. The dose and use of adjuvants depend upon the type and duration of surgery. The armamentarium of adjuvants has been evolving over a while from opioids to several other groups of drugs such as alpha-2 adrenergic receptor agonists, steroids, and anti-inflammatory drugs [4].

Fentanyl, as a novel adjuvant to subarachnoid block, has proven benefits to provide better postoperative analgesia [5,6]. However, nalbuphine, on the other hand, is a newer synthetic opioid with mixed ĸ-receptor agonistic and a partial µ-receptor antagonistic activity, that exhibits its intrathecal analgesic property through the ĸ-receptor [7]. Its safety and efficacy as an adjuvant to local anesthetics in spinal anesthesia remain unproven due to limited studies, and hence, this prospective double-blinded randomized control study was designed to evaluate its potential for postoperative analgesia.

The primary objective of this study is to compare the duration of analgesia between nalbuphine and fentanyl as an intrathecal additive to local anesthetic solutions for postoperative analgesia. The secondary objectives were to compare the quality of sensory and motor blockade and perioperative hemodynamic parameters among the groups.

## Materials and methods

This prospective double-blind randomized controlled study was done to compare postoperative analgesia between nalbuphine and fentanyl as an intrathecal additive in infraumbilical surgeries in the Department of Anesthesiology, Pondicherry Institute of Medical Sciences between January 2021 and December 2022.

Those patients who underwent elective infraumbilical surgeries under spinal anesthesia, belonging to the American Society of Anesthesiologists (ASA) I and II group aged 18-65 years, were included in the trial. We excluded those patients who had raised intracranial pressure or any intracranial pathology, patients on anticoagulants, pregnant women, those with any localized infection, and those allergic to local anesthetics.

Based on the previous study by Prabhakaraiah et al. [8], the mean duration of postoperative analgesia was taken into consideration, and the duration was 180.50 ± 34.58 in group BN (nalbuphine group) and 198.67 ± 45.67 in group BF (fentanyl group) with 80% power and at a 5% level of significance. The sample size required in each group was taken as 79, and 5% dropouts were taken into consideration. The sample size was increased to 83 in each group.

Patients were allocated in a randomized manner by computer-generated randomization chart into two groups of 83 each. Group N (nalbuphine group) received 2.5 mL of 0.5% bupivacaine + 0.5 mL of 1 mg of nalbuphine, and group F (fentanyl group) received 2.5 mL of 0.5% bupivacaine + 0.5 mL of 25 mcg fentanyl intrathecally. A concealed envelope technique was used containing the drug preparation information, which was opened on the day of surgery, and the drug was reconstituted by an anesthesiologist who was not a part of the trial. Both the anesthesiologist involved in the data collection and the patients were blinded to the content of the study solution.

The study was conducted after getting approval from the Institutional Ethics Committee (IEC: RC/2020/89). The trial was registered in the Clinical Trials Registry - India (CTRI) (CTRI/2021/03/031729). After a thorough pre-anesthetic assessment and obtaining written informed consent from the participants, they were enrolled in the study and were premedicated according to routine hospital protocol on the night before surgery. They were kept nil per oral for eight hours before the surgery. The patients were then transferred to the operating room, and standard ASA monitors were attached. Baseline values were recorded. Intravenous access was established, and the infusion of Ringer's lactate was initiated and preloaded with 10-15 mL/kg before spinal anesthesia. All the subarachnoid block was performed by a qualified anesthesiologist in sitting positions at L3-L4 interspace with 2.5 mL of 0.5% hyperbaric bupivacaine + 0.5 mL of either 1 mg nalbuphine or 25 mcg fentanyl. Immediately after administration, the patient was placed in the supine position. The onset of sensory block was assessed using the pinprick method, and motor block was assessed using a modified Bromage score at every two-minute interval. The assessment of sensory block was done by pinprick method in the midline using a 27 G needle every two minutes until the block reached the maximal sensory level.

Grades of sensory blockade

The following are the grades of sensory blockade: grade 0, sharp pain felt; grade 1, dull sensation felt; and grade 2, no sensation felt.

Onset of sensory blockade

The onset of sensory blockade is the interval between the end of the spinal injection and the loss of sensation to pinprick at the T10 dermatome level with a sensory blockade grade of 2.

Peak sensory block

Peak sensory block is achieved when there is no further increase in sensory block after four continuous checks. Time to reach peak sensory level was also noted.

Duration of analgesia

The duration of analgesia is from the onset of sensory blockade until the request of the first rescue analgesic dose.

Modified Bromage score

Motor block was assessed using a modified Bromage score (modified by Breen et al.) [9]. The score criteria are as follows: score 1, complete block (unable to move feet or knees); score 2, almost complete block (able to move feet only); score 3, partial block (just able to move knees); score 4, detectable weakness of hip flexion (between scores 3 and 5); score 5, no detectable weakness of hip flexion while supine; and score 6, able to perform partial knee bend.

Onset of complete motor blockade

The onset of complete motor blockade is the time interval between the completion of the study drug injection until Bromage score 1 was noted.

Duration of motor blockade

The duration of motor blockade is the time between the onset of motor blockade and the return of toe movements.

Two-segment regression

Two-segment regression is the time interval between intrathecal administration of the drug and the time to regress two dermatomal segments from the peak sensory block.

If adequate sensory and motor blockade was not achieved after 30 minutes from the administration of spinal blockade, then the block was considered a failure, and the patients were excluded from the study and were converted into general anesthesia. Hemodynamic parameters such as heart rate, blood pressure (systolic and diastolic), and oxygen saturation (SpO2) were monitored continuously and recorded at 5, 10, 15, 20, 25, and 30 minutes after the spinal injection and subsequently every 15 minutes until there were two segments of regression, and this time, it was noted by pinprick method. The time for complete recovery of motor block was also noted. Adverse events such as hypotension, bradycardia, pruritus, nausea, and vomiting were also noted.

Intraoperative hypotension was defined as the decrease in systolic blood pressure by 20% from the baseline, and it was treated with injection of ephedrine 6 mg IV boluses as appropriate. Bradycardia was defined as a heart rate of less than 50/minute and was treated with an injection of atropine 0.6 mg IV. Hypoxia was defined as desaturation < 90% and was supplemented with oxygen. Pruritus was treated with antihistamines, and nausea and vomiting with antiemetics. Postoperative pain was assessed using the visual analog scale (VAS), which was explained to the patient during a pre-anesthetic checkup (PAC). If the VAS score is >3, rescue analgesia (injection diclofenac 75 mg IV) was given, and this was also noted. Postoperatively, patients were followed up for the next 24 hours to look for any side effects, which were noted down if any.

For statistical analysis, all data were expressed as mean ± standard deviation. Data were entered in Microsoft Excel (Microsoft Corp., Redmond, WA), and analysis was done using Statistical Package for the Social Sciences (SPSS) version 2.0 (IBM SPSS Statistics, Armonk, NY). To represent categorical data, frequency and percentage were used. Qualitative data were presented as mean and standard deviation. The Chi-square test was used to find an association between categorical data. Student's unpaired t-test was used to find an association between the quantitative data if data followed normality. For non-normal distribution, the Mann-Whitney U test was used. P value < 0.005 was considered significant. Appropriate graphs such as bar charts and line diagrams were used to represent the data.

## Results

**Figure 1:**
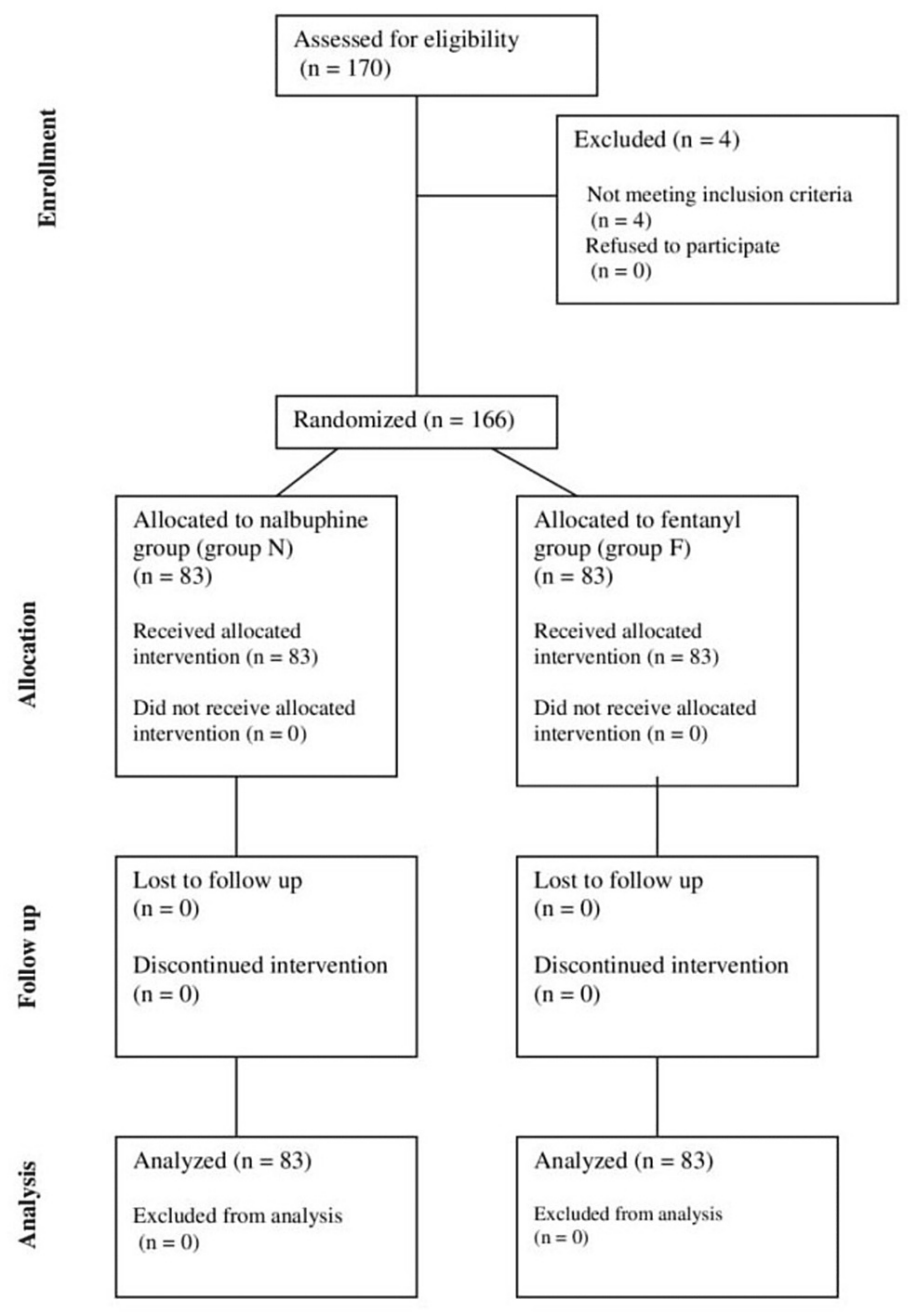
CONSORT flowchart Group N: nalbuphine, group F: fentanyl, CONSORT: Consolidated Standards of Reporting Trials

A total of 166 patients aged between 18 and 60 years belonging to ASA I and II and those who underwent infraumbilical surgeries were randomly allocated into two groups, group N and group F, of 83 patients each (Figure 1).

As seen in Table 1, the mean age distribution among group N was 45.23 ± 8.289 years, and in group F, it was 45.94 ± 6.698 years, with a P value of 0.019. The mean gender distribution in group N was 29, and in group F, it was 137, with a P value of 0.838. The mean weight distributions among the groups were 70.42 ± 11.507 kg in group N and 72.34 ± 12.575 kg in group F, with a P value of 0.307. The mean height distribution was 165.71 ± 4.528 in group N and 163.17 ± 4.894 in group F, with a P value of 0.736. The ASA I and II were taken into consideration, and there were 39 and 33 ASA I patients and 44 and 50 ASA II patients in group N and group F, respectively, with a P value of 1.0. The results were comparable among the two groups.

**Table 1:** Demographic data Group N: nalbuphine, group F: fentanyl, kg: kilogram, cm: centimeter, ASA: American Society of Anesthesiologists

Variables		Group N (n = 83)	Group F (n = 83)	P value
Age (years)		43.63 ± 12.000	47.83 ±10.824	0.019 (Student's unpaired t-test)
Gender	Male	14 (8.4%)	15 (9%)	0.838 (Chi-square test)
	Female	69 (41.6%)	68 (41%)	
Weight (kg)		70.42 ± 11.507	72.34 ±12.575	0.307 (Student's unpaired t-test)
Height (cm)		165.71 ± 4.528	163.17 ± 4.894	0.736 (Student's unpaired t-test)
ASA	I	39 (23.5%)	33 (19.9%)	1.0 (Chi-square test)
	II	44 (26.5%)	50 (30.1%)	

Based on Table 2, there was a significant difference in the following parameters: onset of sensory blockade (mean time taken to achieve T10), which was 3.94 ± 1.7 69 minutes in group N and 5.94 ± 0.929 minutes in group F, with a P value of <0.001; mean time taken to achieve peak sensory block level (PSBL), which was 6.22 ± 1.970 minutes in group N and 9.81 ± 0.903 minutes in group F, with a P value of <0.001; onset of complete motor blockade (time taken to achieve M1), which was 7.10 ± 1.858 minutes in group N and 11.61 ± 1.218 minutes in group F, with a P value of <0.001; mean duration of motor blockade between the two groups, which was 182.57 ± 13.011 minutes in group N and 112.53 ± 7.389 minutes in group F, with a P value of <0.001; and duration of analgesia between the two groups, which was 267.27 ± 172.099 minutes in group N and 161.35 ± 14.957 minutes in group F, with a P value of <0.001. However, the PSBL achieved in group N was T4 in 13 patients, T6 in 26 patients, and T8 in 44 patients, whereas in group F, T4 was achieved in one patient, T6 was achieved in 29 patients, and T8 was achieved in 53 patients, with a P value of 0.004. The mean time taken for two-segment regression among the two groups was 118.20 ± 12.61 minutes in group N and 113.72 ± 8.84 minutes in group F, with a P value of 0.009. The results were comparable.

**Table 2:** Block characteristics, onset, and regression time Group N: nalbuphine, group F: fentanyl, PSBL: peak sensory block level, mins: minutes, *: statistically significant, T4: loss of sensation at the level of the nipple, T6: at the level of xiphoid, T8: four finger breadth from the umbilicus

Characteristics		Group N (n = 83)	Group F (n = 83)	P value
Time to achieve T10/onset of sensory blockade (mins)		3.94 ± 1.769	5.94 ± 0.929	<0.001* (Student's unpaired t-test)
PSBL achieved (n)	T4	13 (7.8%)	1 (0.6%)	0.004 (Chi-square test)
	T6	26 (15.7%)	29 (17.5%)	
	T8	44 (26.5%)	53 (31.9%)	
Time to achieve PSBL (mins)		6.22 ± 1.970	9.81 ± 0.903	<0.001* (Student's unpaired t-test)
Two-segment regression time (mins)		118.20 ± 12.61	113.72 ± 8.84	0.009 (Student's unpaired t-test)
Time to achieve M1/onset of motor blockade (mins)		7.10 ± 1.858	11.61 ± 1.218	<0.001* (Student's unpaired t-test)
Duration of motor blockade (mins)		182.57 ± 13.011	112.53 ± 7.389	<0.001* (Student's unpaired t-test)
Time to rescue analgesia (mins)		267.27 ± 172.099	161.35 ± 14.957	<0.001* (Mann-Whitney U test)

**Figure 2:**
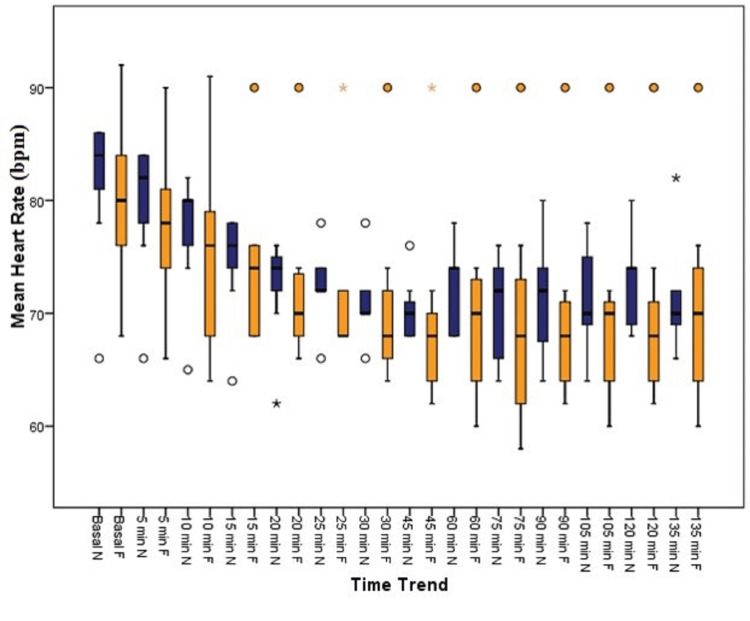
Distribution of heart rate among the groups Min: minutes, bpm: beats per minute, N: nalbuphine, F: fentanyl

As seen in Figure 2, heart rate clinically differed in group N at 20, 25, 30, and 45 minutes compared to group F, but it was not statistically significant.

**Figure 3:**
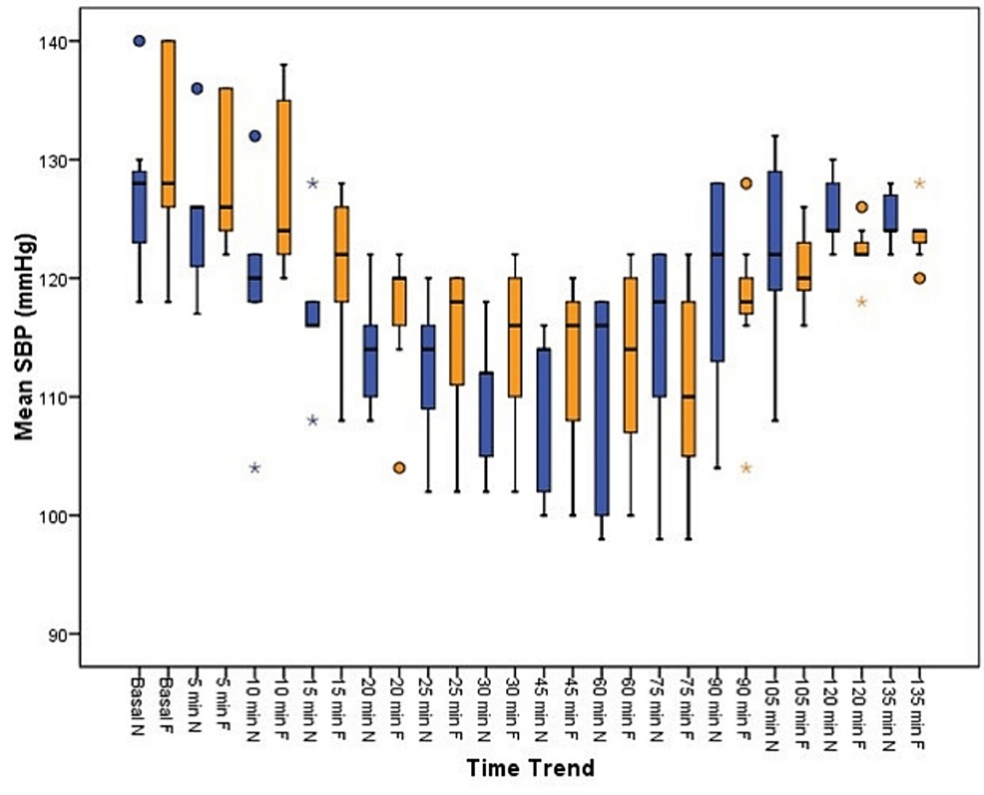
Distribution of SBP among the groups SBP: systolic blood pressure, mmHg: millimeters of mercury, min: minutes, N: nalbuphine, F: fentanyl

Based on Figure 3, there was no statistically significant difference in systolic blood pressure at any time period between the two groups. 

**Figure 4:**
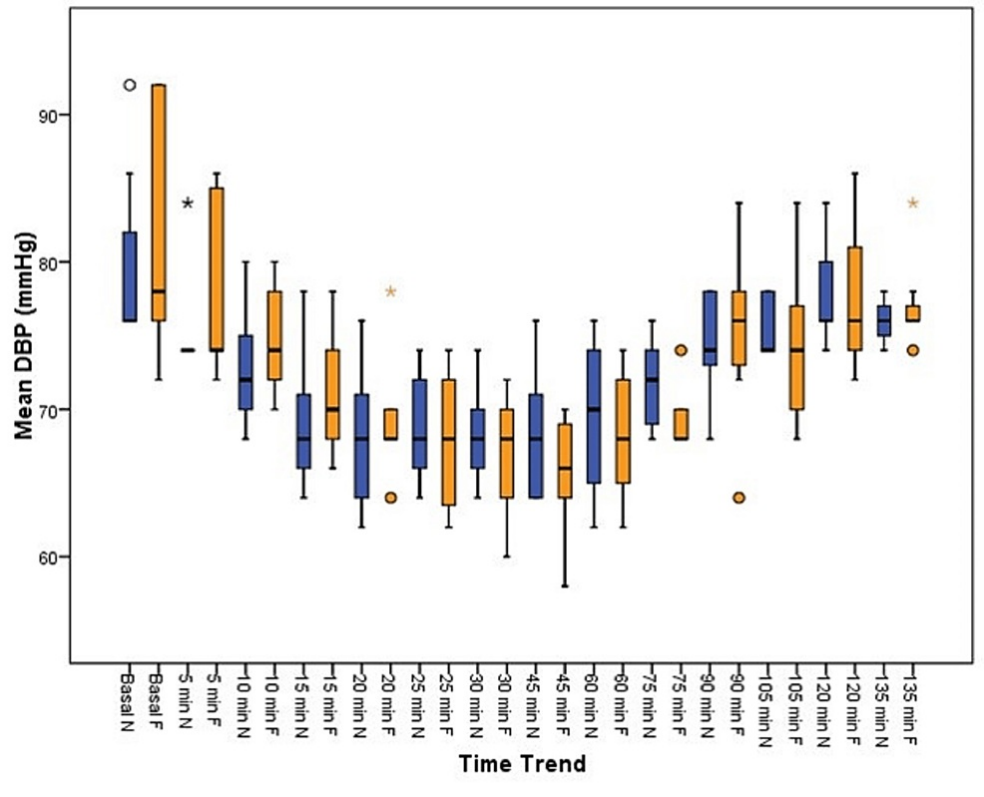
DBP distribution among the groups DBP: diastolic blood pressure, mmHg: millimeters of mercury, min: minutes, N: nalbuphine, F: fentanyl

Based on Figure 4, there was no statistically significant difference in the diastolic blood pressure at any time period between the two groups.

## Discussion

Spinal anesthesia is the most preferred mode of anesthesia for infraumbilical surgeries. Intrathecal adjuvants were used along with local anesthetics to prolong the duration of anesthesia and provide postoperative analgesia, thereby minimizing the dose of local anesthetic agents. Opioids such as fentanyl, morphine, and buprenorphine are routinely used adjuvants to local anesthetic agents [10]. Fentanyl is a preferential μ-receptor agonist that has proven benefits to provide better postoperative analgesia as an adjuvant to subarachnoid block, thus facilitating the reduction in the dose of local anesthetics [11]. On the other hand, nalbuphine, which is a μ-receptor antagonist and ĸ-receptor agonist, has been used as an adjuvant in recent times. It has the potential to provide good intra- and postoperative analgesia with decreased incidence and severity of μ-receptor side effects [7]. This double-blind randomized controlled study was conducted to compare the postoperative analgesic efficacy between intrathecal nalbuphine and intrathecal fentanyl along with local anesthetics in spinal anesthesia.

Demographic data such as age, gender, height, weight, and ASA grading (I/II) distribution between the groups were comparable.

The mean duration of analgesia according to our observations was 267.27 ± 172.099 minutes in group N and 161.35 ± 14.957 minutes in group F, which was both clinically and statistically significant, with a P value of <0.001. This implies that the duration of analgesia during the postoperative period was comparatively both clinically and statistically better in the nalbuphine group in comparison with the fentanyl group. Similar results were obtained by Garg et al. [12] and Gupta et al. [13] who inferred that the mean time for the first rescue analgesic request in the nalbuphine group was significantly better when compared to the fentanyl group. However, there were a few divergences from our observations from Srinivasaiah et al. [14] and Gomaa et al. [15] who found no significant difference in the duration of analgesia between nalbuphine and fentanyl. The main presumption for such a divergence in findings could be attributed to a lesser dose of nalbuphine employed in both trials in contrast to us.

The onset of sensory blockade (mean time to achieve T10) was significantly faster in the nalbuphine group (3.94 ± 1.769 minutes) than in the fentanyl group (5.94 ± 0.929 minutes), with a P value of <0.001. Similar observations were iterated by Shalini et al. [16], demonstrating a significantly quicker onset when adding nalbuphine to the mixture compared to clonidine, whose findings are well in line with our inferences. Also, there was a significant difference in the mean time taken to achieve peak sensory block level in group N (6.22 ± 1.970 minutes) compared to group F (9.81 ± 0.903 minutes), with a P value of <0.001. On the contrary, there is other evidence to suggest that fentanyl might have quicker onset times with sensory blockade than nalbuphine possibly due to its higher lipid solubility and its ability to cross the blood-brain barrier more freely [17,18].

Nalbuphine could undoubtedly achieve peak sensory blockade much earlier than fentanyl but did not perform better in terms of two-segment regression. With regression times of 118.20 ± 12.610 minutes with nalbuphine compared to 113.72 ± 8.840 minutes with fentanyl, there existed a clinical but not statistical difference between the two according to our observations. Nevertheless, there are instances where nalbuphine outlasted fentanyl in terms of two-segment regression as outlined by Bisht et al. [19], who demonstrated two-segment regression times of 97.72 ± 9.5 minutes in the nalbuphine group against 88.8 ± 9.4 minutes in the fentanyl group, with the difference being statistically significant.

Nalbuphine's performance in the front of motor onset had been astonishing as it could achieve motor onsets much earlier and statistically significant compared to fentanyl with onset times of 7.10 ± 1.86 minutes in group N versus 11.61 ± 1.218 minutes in group F, with a P value of <0.001. In contrast, Mavaliya et al. [20] observed similar onset times between nalbuphine and fentanyl groups (7.14 ± 1.03 versus 6.97 ± 0.95 minutes), with insignificant P values of 0.457. This change in observation could possibly be attributed to their use of ropivacaine as a local anesthetic, which would otherwise possess a preferential sensory-blocking capacity.

Nalbuphine also faired supremely well in terms of duration of motor blockade, depicting duration times significantly outlasting those of the fentanyl group in this study (182.57 ± 13.01 minutes (group N) versus 112.53 ± 7.39 minutes (group F)). These observations were in disagreement with Naaz et al. [21], who found the duration of motor blockade in the nalbuphine group at a dose of 0.8 mg and 1.6 mg to be comparable with the fentanyl group at a dose of 25 mcg, with a P value of 0.456. However, in the present study, there was a statistically significant difference in the mean duration of motor blockade in the nalbuphine group, with a P value of <0.001. This also firmly establishes our findings that the faster peak sensory and motor onset times and prolonged duration of motor blockade could all be related to the higher dose of intrathecal nalbuphine incorporated in this study, mindfully eliminating the adverse neuraxial side effects aiming for an optimal dose.

Gurunath et al. [22] observed a lower heart rate in the nalbuphine group compared to the fentanyl group, with significant differences at 6, 9, and 12 minutes, whereas the present study cohorts differed in their heart rates clinically at 25, 30, and 45 minutes, but no statistical difference could be derived. Hemodynamic parameters such as systolic blood pressure and diastolic blood pressure were all comparable between the groups from baseline until the end of the procedure, with insignificant P values, a finding well in agreement with the finding of Garg et al. [12].

To reiterate the safety profile of 1 mg of nalbuphine in the intrathecal space, none of the study cohorts had any side effects in the form of pruritus, nausea, or vomiting in this study compared to reported occurrences of pruritus in the fentanyl group observed by Prabhakaraiah et al. [8].

There were a few limitations to our study, where intragroup analyses on the type of surgery and its effects on hemodynamic parameters were not included. Also, the optimal dose of nalbuphine with multigroup analysis was not done, which could be an area of caveat for further research.

## Conclusions

Considering the more prolonged duration of analgesia, intrathecal nalbuphine in the postoperative period was found to be more safe and effective than intrathecal fentanyl when used as an adjuvant therapy with 0.5% hyperbaric bupivacaine in patients undergoing elective infraumbilical surgeries with hemodynamic stability and no side effects.
